# Down-regulation of miR-340-5p promoted osteogenic differentiation through regulation of runt-related transcription factor-2 (RUNX2) in MC3T3-E1 cells

**DOI:** 10.1080/21655979.2021.1905259

**Published:** 2021-04-05

**Authors:** Xiaochen Wang, Yaochuan Mi, Wei He, Xiaona Hu, Shuo Yang, Lu Zhao, Yanyang Zhang, Binhong Wen

**Affiliations:** a Department of Endocrinology, The People’s Hospital of China Medical University, The People’s Hospital of Liaoning Province, Shenyang, LiaoningP.R. China; b Department of Endocrinology, Dalian Medical University, Dalian, Liaoning, P.R. China

**Keywords:** Mir-340-5p, osteogenic differentiation, runx2, mc3t3-e1 cells

## Abstract

Diabetic osteoporosis (DOP) is a chronic complication of diabetes in the skeletal system. High level of miR-340-5p may be harmful to the bone formation. In this study, the DOP model of rats was successfully established via streptozotocin (STZ) and ovariectomy (OVX) treatment. It was manifested by reduced body weight, insulin level, alkaline phosphatase (ALP) activity, and osteocalcin (OCN) and collagen-I expressions, as well as increased concentration of fasting blood glucose. Moreover, we found that miR-340-5p expression was increased while runt-related transcription factor-2 (RUNX2) was decreased in femurs. Furthermore, the effects of miR-340-5p on osteogenic differentiation (OD) in high glucose (HG)-treated MC3T3-E1 cells were explored. Exposure to OD and HG contributed to elevated miR-340-5p level. Inhibition of miR-340-5p enhanced ALP level, calcium deposition, and OCN, collagen-I and RUNX2 levels. On the contrary, miR-340-5p overexpression reversed these promotional effects. Luciferase assay indicated that RUNX2 may be a target gene of miR-340-5p. Moreover, RUNX2 deficiency decreased miR-340-5p inhibition-induced ALP activity, calcium accumulation and OCN, collagen-I, RUNX2 levels. In short, the above findings revealed that inhibition of miR-340-5p facilitated osteogenic differentiation through regulating RUNX2 in MC3TC-E1 cells, which provided targeted therapeutic strategies for the treatment of DOP.

## 1. Introduction

Diabetes is a chronic and metabolic disease, characterized by elevated blood glucose concentration due to insulin deficiency or insulin resistance [1]. The occurrence of diabetes is usually concomitant with multiple complications, including microvascular disease, nephropathy, and osteopathy [2]. Diabetic osteoporosis (DOP) refers to a chronic complication of diabetes in the skeletal system, and is manifested by decreased bone mineral density (BMD), enhanced bone fragility and increased risk in fracture [3,4]. Osteoporosis is caused by the imbalance between osteoclastic bone resorption and osteoblastic bone formation. Reducing bone resorption and increasing bone formation are the most basic and effective measures for the treatment of osteoporosis. At present, most anti-osteoporotic drugs such as bisphosphonates, denosumab, and thiazolidinediones affect bone metabolism and increase the risk of fracture [5,6]. Therefore, it is essential to seek novel therapeutic strategies that can promote osteoblast differentiation and enhance bone formation, thereby attenuating DOP.

MicroRNAs (miRNAs) are a class of small non-coding RNAs that bind to target genes participating in a variety of biological processes, including organ development, cell proliferation, differentiation, and apoptosis [7,8]. For example, miR-532-3p inhibited osteogenic differentiation (OD) in MC3T3-E1 cells by reducing E-26 transformation specific-1 (ETS1) [9]. MiR-19a-3p facilitated osteoblast differentiation of human-derived mesenchymal stem cells (hMSCs) through decreasing HDAC4 [10]. A study indicated that miR-340-5p was elevated in response to inflammatory cytokine-stimulated β cell injury, implying the association between miR-340-5p and type I diabetes [11]. Besides, Du et al. pointed out that miR-340-5p inhibition facilitated osteogenesis of bone marrow-derived mesenchymal stem cells (BMSCs) [12]; however, the effect of miR-340-5p on osteoblast differentiation has not been fully clarified.

Runt-related transcription factor-2 (RUNX2), a nuclear transcription factor, has been reported to exert a critical role in osteoblast differentiation and bone development [13]. Several researches demonstrated that RUNX2 expression was down-regulated in osteoporosis, and it was considered to be a typical marker of osteoporosis [5,14,15]. Moreover, RUNX2 was found to be involved in the regulation of BMSCs differentiation [16]. Bioinformatics website predicted that miR-340-5p may target RUNX2 3ʹ untranslated region (UTR). Therefore, we hypothesized that miR-340-5p/RUNX2 might play an important role in the process of osteoblast differentiation.

In the current study, the role of miR-340-5p in osteoblast differentiation was investigated. We established the rat model of DOP and MC3T3-E1 cell model of inhibiting osteoblast differentiation. It was shown that miR-340-5p expression was increased in model groups. In addition, the lowered miR-340-5p level promoted osteoblast differentiation via up-regulation of RUNX2 from following aspects: alkaline phosphatase (ALP) detection, Alizarin Red staining, and measurement of OD-related factors including osteocalcin (OCN), collagen-I, as well as RUNX2. This finding demonstrated that inhibition of miR-340-5p contributed to osteoblast differentiation by regulating RUNX2 in MC3TC-E1 cells, providing some references for preventing and curing DOP.

## 2. Materials and methods

### 2.1. The rat model of DOP

All procedures on the animals were approved by the Institutional Animal Care and Use Committee of The People’s Hospital of Liaoning Province (Shenyang, China). Eight-week-old healthy and female Sprague-Dawley (SD) rats weighing 180–220 g were housed under standard circumstances (12-h light/dark cycle, 22 ± 1°C, humidity of 45–55%, with access to food and water *ad libitum*) for a week. All the rats were randomly assigned into four groups (N = 6 rats in each group): sham, streptozotocin (STZ), ovariectomy (OVX), and STZ+OVX.

After fasting overnight, a diabetic model was conducted via intraperitoneal injection of STZ (S110910, Aladdin, China) at a dose of 60 mg/kg body weight [6]. Meanwhile, the rats in sham and OVX groups received an equal volume of solvent. 72 h later the concentrations of fasting blood glucose were measured, Mediating diabetes was successful when glucose content was higher than 16.7 mM. Subsequently, bilateral OVX in rats was carried out based on the previous description of Lasota et al. [17]. The rats were fed for 8 weeks, and the body weight was recorded once a week. Besides, the animals were fasted overnight, and then fasting blood glucose and blood insulin contents were assessed using the commercial kits (F006, Nanjing Jiancheng Bioengineering Institute, China; CEA448Ra, USCN KIT INC., China). Finally, all the animals were sacrificed and the femur tissues were collected for following experiments.

### 2.2. Determination of Bone Mineral Density (BMD)

The bone mineral density (BMD) at the proximal femurs was determined using a Lunar Version 4.7e DXA bone densitometer (GE, USA). The values were presented as the number of grams of bone mineral per square millimeter (g/mm [2]).

### 2.3. ALP staining

The distal third of the femur was selected as the region of interest for detection of ALP levels. The samples were embedded in paraffin and cut into 5-μm sections. Then slices were deparaffinated, dehydrated, and stained with ALP staining solution (DE0001, Leagene Biotechnology, China) for 12 h at 37°C. After washing with running water, the sections were counterstained with hematoxylin (H8070, Solarbio, China) for 5 min, and observed under a microscope (BX53, OLYMPUS, Japan).

### 2.4. Cell culture and induction

Mouse embryonic osteoblasts MC3T3-E1 (clone 14) were purchased from Cell Resource Center, Shanghai Institutes for Biological Sciences, Chinese Academy of Sciences (China). Cells were cultured in DMEM (31,600–034, Gibco, USA) supplemented with 10% FBS (F8067, Sigma, USA) under standard conditions (37°C, 5% CO_2_).

To sufficiently induce osteoblast differentiation, when the cell confluence reached about 90%, they were incubated with 5.5 mM glucose (G0350500, Sigma), 5 mM β-glycerophosphate (G9422, Sigma), 10^−^[7] M dexamethasone (D4902, Sigma), and 50 mg/mL ascorbic acid (A103539, Aladdin). In OD+high glucose (OD+HD) group, cells were treated with 25 mM glucose, and other reagents were the same as OD group. Meanwhile, control cells were administrated by 5.5 mM glucose. After incubation for 14 days, a series of assays were conducted.

### 2.5. Cell infection and stimulation

On the one hand, to investigate the role of miR-340-5p in DOP *in vitro*, cells were infected with lentivirus-based miR-340-5p inhibitor (anti-miR-340-5p), pre-mir-340 (miR-340-5p) or their negative controls (anti-NC and NC) for 72 h [18,19]. Subsequently, *in vitro* differentiation was induced, and cells were treated with HG for 14 days. On the other hand, to evaluate the effect of RUNX2, the cells were infected with lentivirus-mediated RUNX2 shRNA (shRUNX2) or NC shRNA for 72 h, and the efficiency was determined. They were then co-infected with anti-miR-340-5p or anti-NC and shRUNX2 or NC shRNA for 72 h. The OD and HG induction was carried out in consistent with the above steps.

### 2.6. Luciferase assay

293 T cells (Shanghai Zhong Qiao Xin Zhou Biotechnology Co., Ltd., China) were cultured with DMEM containing 10% FBS. Cells were co-transfected with miR-340-5p mimic or NC mimic and RUNX2 3ʹ UTR wild type (WT) or RUNX2 3ʹ UTR mutant type (MT) for 48 h. After co-transfection, cells were lysed, and the luciferase activity was evaluated by the kit (KGAF040, KeyGEN BioTECH, China) according to the manufacturer’s instructions.

### 2.7. Detection of ALP activity

The level of ALP was assessed via the kit (A059, Nanjing Jiancheng Bioengineering Institute) in accordance with the manufacturer’s recommendation. In brief, 200 μL of PBS was added to treated cells, and ultrasonication was conducted on ice. After centrifugation (2500 rpm, 10 min), the supernatants were collected as samples. ALP level was detected according to the standard protocol. The absorbance at 520 nm was measured by a microplate reader (ELX-800, BIOTEK, USA).

### 2.8. Alizarin Red staining

Treated cells were rinsed with PBS twice and fixed in 4% paraformaldehyde. Cells were stained with Alizarin Red staining solution (G1450, Solarbio) for 20 min. After being washed, calcium nodules were imaged via a microscope. For quantitative analysis, 10% CPC (1,104,006, Sigma) was added to cells. After reaction for 15 min, the absorbance at 570 nm was measured by a microplate reader.

### 2.9. Quantitative real-time PCR (qRT-PCR)

Total RNA was extracted from rat femur tissues or cells using TRIpure (RP1001, BioTeke, China). RNA was reversely transcribed into cDNA utilizing Super M-MLV Reverse Transcriptase (PR6502, BioTeke). For miRNA quantification, qRT-PCR was conducted with SYBR Green (S9430, Sigma) on an Exicycler 96 PCR instrument (BIONEER, Korea). 5S was acted as the internal reference. The primers were synthesized by Genscript Biotechnology Co., Ltd (China), and their sequences were as below: mmu/rno-miR-340-5p (MIMAT0004651/MIMAT0004650; F: 5ʹ-TTATAAAGCAATGAGACTGATT-3ʹ, R: 5ʹ-GCAGGGTCCGAGGTATTC-3ʹ), 5S (rat) (NR_033176.2; F: 5ʹ-GATCTCGGAAGCTAAGCAGG-3ʹ, R: 5ʹ-TGGTGCAGGGTCCGAGGTAT-3ʹ), and 5S (mouse) (NR_03 0686.1; F: 5ʹ-CTAAAGATTTCCGTGGAGAG-3ʹ, R: 5ʹ-TGGTGCAGGGTCCGAGGTAT-3ʹ).

### 2.10. Immunoblotting

Proteins were extracted from rat femur tissues or cells with lysis solution (P0013, Beyotime, China) containing 1% PMSF (ST506, Beyotime). The protein concentration was determined by the BCA kit (P0011, Beyotime). After denaturation and SDS-PAGE electrophoresis, separated proteins were transferred onto PVDF membranes (IPVH00010, Millipore, USA). The membranes were blocked with 5% nonfat milk in TBST for 1 h, and incubated overnight at 4°C with the primary antibodies against OCN (DF12303, 1:500, Affinity, China), collagen-l (AF0134, 1:500, Affinity), RUNX2 (AF5186, 1:1000, Affinity), and β-actin (sc-4778, 1:1000, Santa Cruz, USA). After washing with TBST, the membranes were incubated for 45 min at 37°C with secondary antibodies including HRP-labeled goat anti-rabbit IgG (A0218, 1:5000, Beyotime) and HRP-labeled goat anti-mouse IgG (A0216, 1:5000, Beyotime). The immunoreactive protein was visualized by a WD-9413B gel imaging system (Beijing Liuyi, China) with ECL kit (P0018, Beyotime).

### 2.11. Statistical analysis

Data were expressed as means ± standard deviation (SD). Statistical differences among different experimental groups were assessed by one-way ANOVA and two-way ANOVA utilizing GraphPad Prism 8. A value of *P* < 0.05 was considered significant.

## 3. Results

### 3.1. The rat model of diabetic osteoporosis (DOP) was established

**Figure 1.**
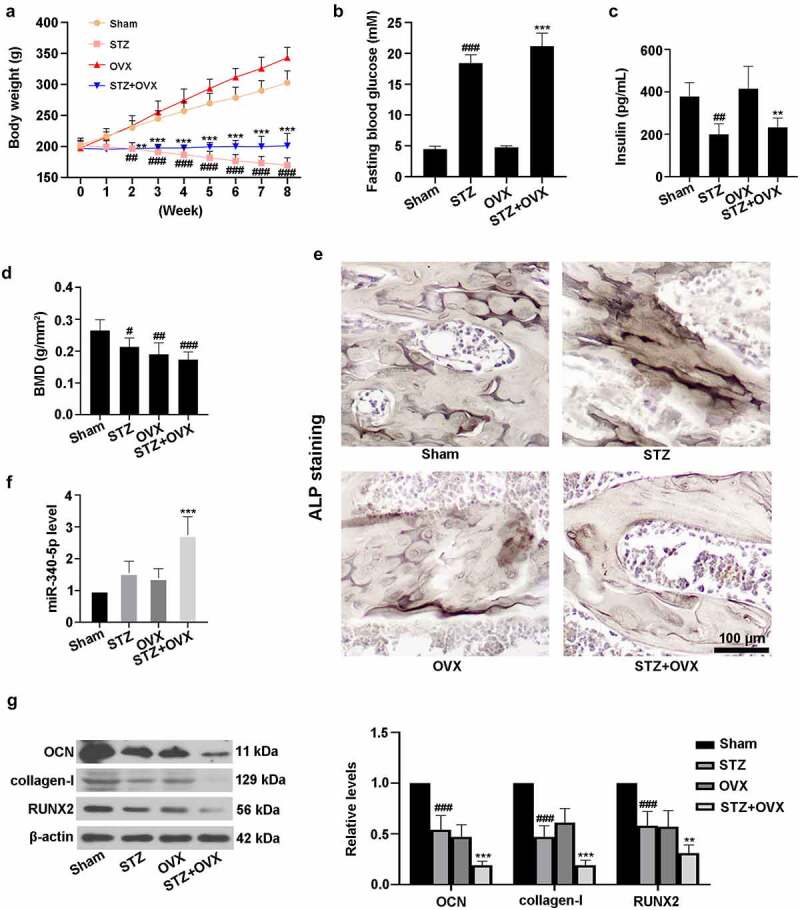
The rat model of diabetic osteoporosis was established. (a-c) Diabetes was mediated by STZ in rats. Subsequently, bilateral OVX was carried out. The rats were fed for 8 weeks, and body weight was detected once a week. Then the animals were fasted overnight, and fasting blood glucose and blood insulin contents were assessed using the commercial kits. Finally, all the animals were sacrificed, and rat femur tissues were collected for following experiments. (d) Detection of BMD. (e) ALP staining was performed in femur tissues. Scale bar = 100 μm. (f) Measurement of miR-340-5p expression by qRT-PCR. (g) Evaluation of OCN, collagen-I, and RUNX2 levels with immunoblotting. β-actin was used as the internal reference. STZ, streptozotocin; OVX, ovariectomy; BMD, bone mineral density; ALP, alkaline phosphatase; OCN, osteocalcin. Data were expressed as means ± SD (N = 6 per group). ^#^*P* < 0.05, ^##^*P* < 0.01, and ^###^*P* < 0.001 versus sham group; **P < 0.01 and ***P < 0.001 versus OVX group

To establish a DOP model *in vivo*, the rats were subjected to STZ injection and OVX. As shown in Figure 1(a), the body weight of rats in STZ group was significantly lower than that of sham rats from week 2 to week 8. Similarly, STZ+OVX administration also decreased the rat body weight when compared with OVX rats. Figure 1(b) indicated that STZ treatment increased the levels of fasting blood glucose in rats of STZ or STZ+OVX groups. Also, the lowered insulin contents were observed in STZ and OVX+STZ groups (Figure 1(c)). Notably, compared with sham rats, the value of BMD was markedly reduced among the other three groups (Figure 1(d)). ALP staining revealed that ALP activity was elevated in STZ rats, while being decreased in STZ+OVX rats (Figure 1(e)). In addition, Figure 1(f) suggested that miR-340-5p level was significantly up-regulated in femurs of rats with STZ and OVX treatment. As depicted in Figure 1(g), STZ and OVX induced the reduction of OCN, collagen-I, and RUNX2 protein expressions. The above results demonstrated that establishment of rat model of DOP was successful, and miR-340-5p may play a key role in DOP.

### 3.2. High glucose inhibited osteogenic differentiation in MC3T3-E1 cells

**Figure 2.**
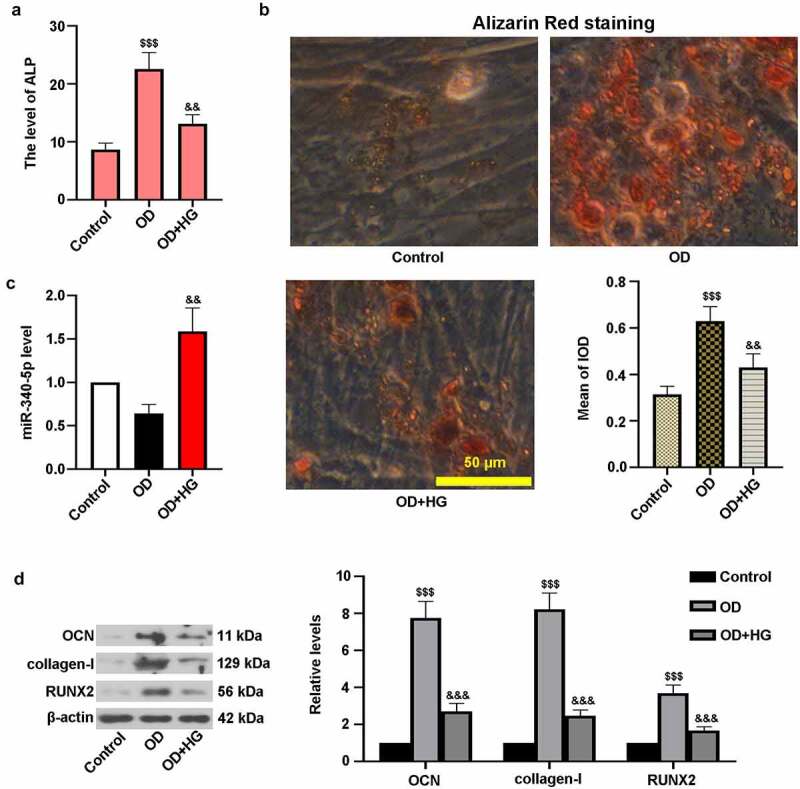
High glucose inhibited osteogenic differentiation in MC3T3-E1 cells. osteoblast differentiation was induced in cells, and cells were treated with HG for 14 days. (a) ALP activity was measured with the kit. (b) Treated cells were stained with Alizarin Red and quantified accordingly. Scale bar = 50 μm. (c) The level of miR-340-5p was detected by qRT-PCR. (d) OCN, collagen-I, and RUNX2 protein levels were assessed with immunoblotting. OD, osteogenic differentiation; HG, high glucose; IOD, integrated optical density. Results were presented as means ± SD (N = 3 in each group). ^$$$^*P* < 0.001 versus control group; ^&&^*P* < 0.01 versus OD group

To induce DOP in MC3T3-E1 cells, they were treated with OD and HG for 14 days. Figure 2(a) showed that HG treatment reduced the level of ALP in cells. It can be seen from Figure 2(b) that calcium accumulation in OD group was elevated in comparison with control cells. Nevertheless, HG resulted in the decrease of calcium deposition. Moreover, high level of miR-340-5p was observed in OD and HG-administrated cells (Figure 2(c)). Figure 2(d) showed that the protein levels of OCN, collagen-I, and RUNX2 were lower in OD+HG group than that of OD-treated cells. These findings suggested that HG inhibited osteoblast differentiation in MC3T3-E1 cells.

### 3.3. Down-regulation of miR-340-5p promoted osteogenic differentiation in MC3TC-E1 cells

**Figure 3.**
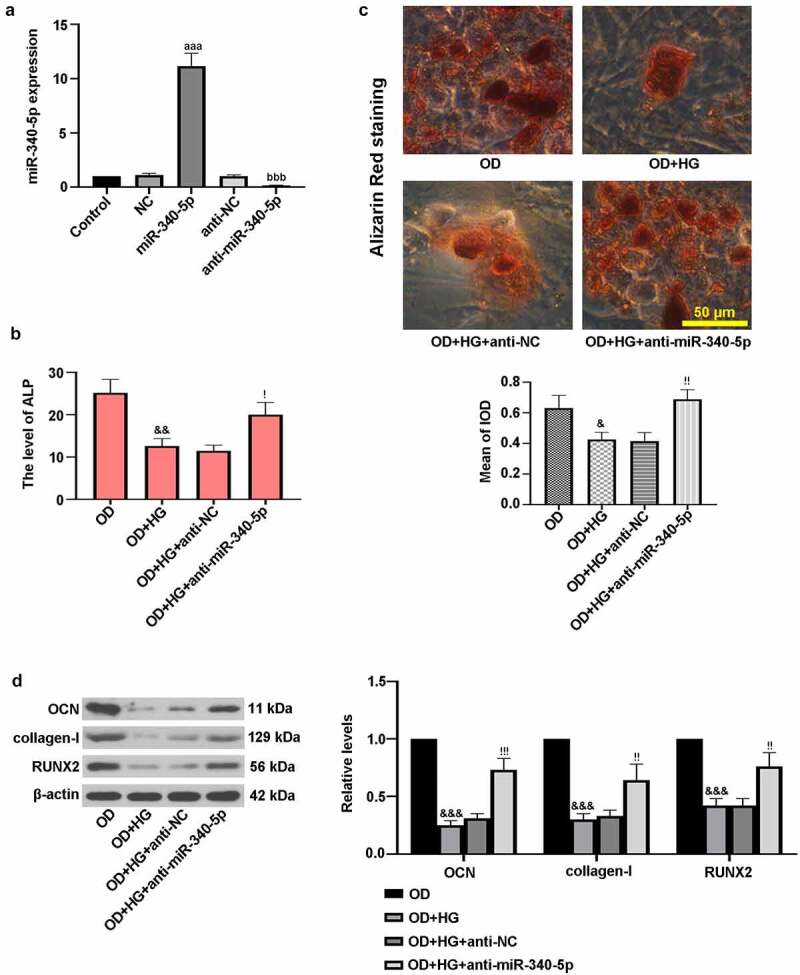
Down-regulation of miR-340-5p promoted osteogenic differentiation in MC3T3-E1 cells. (a) Cells were infected with lentivirus-based miR-340-5p inhibitor (anti-miR-340-5p), pre-mir-340 (miR-340-5p) or their negative controls (anti-NC and NC) for 72 h. Subsequently, osteoblast differentiation was induced, and cells were treated with HG for 14 days. (b) ALP activity was measured with the kit. (c) Treated cells were stained with Alizarin Red and quantified accordingly. Scale bar = 50 μm. (d) OCN, collagen-I, and RUNX2 protein levels were assessed with immunoblotting. NC, negative control. Data were expressed as means ± SD (N = 3 per group). ^aaa^*P* < 0.001 versus NC group; ^bbb^*P* < 0.001 versus anti-NC group; ^&^*P* < 0.05 and ^&&^*P* < 0.01 versus OD group; ^!^*P* < 0.05, ^!!^*P* < 0.01 and ^!!!^*P* < 0.001 versus OD+HG+anti-NC group

MC3T3-E1 cells were infected with miR-340-5p, anti-miR-340-5p and their controls, and the efficiency was determined by RT-qPCR assay. As shown in Figure 3(a), miR-340-5p expression was significantly decreased by anti-miR-340-5p treatment, while it was increased following miR-340-5p treatment. Further, to examine the roles of miR-340-5p in osteoblast differentiation, cells were then treated with OD+HG. Figure 3(b) suggested that ALP level was elevated by miR-340-5p inhibition in OD+HG-administrated cells. As displayed in Figure 3(c), miR-340-5p loss significantly increased the deposition of calcium. Additionally, miR-340-5p inhibition up-regulated the expression levels of OCN, collagen-I, and RUNX2 (Figure 3(d)). The above results confirmed that inhibition of miR-340-5p facilitated osteoblast differentiation in MC3TC-E1 cells treated with HG.

### 3.4. Overexpression of miR-340-5p blocked osteogenic differentiation in MC3TC-E1 cells

**Figure 4.**
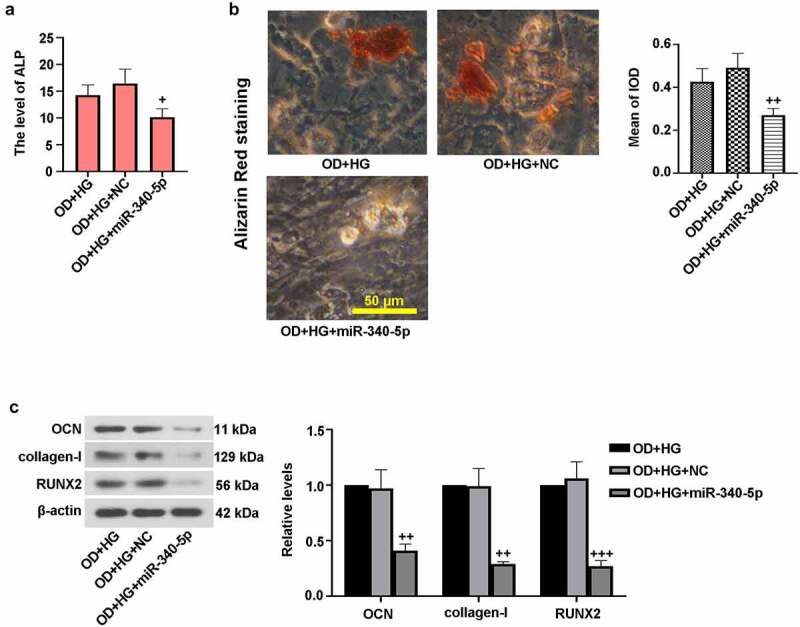
Up-regulation of miR-340-5p blocked osteogenic differentiation in MC3T3-E1 cells. MiR-340-5p was overexpressed (miR-340-5p) via lentivirus infection. Subsequently, osteoblast differentiation was induced, and cells were treated with HG for 14 days. (a) ALP activity was measured with the kit. (b) Treated cells were stained with Alizarin Red and quantified accordingly. Scale bar = 50 μm. (c) OCN, collagen-I, and RUNX2 protein levels were assessed with immunoblotting. Data were indicated as means ± SD (N = 3 per group). ^+^*P* < 0.05, ^++^*P* < 0.01 and ^+++^*P* < 0.001 versus OD+HG+NC group

To further confirm the effect of miR-340-5p on osteoblast differentiation, MC3T3-E1 cells were infected with overexpressed miR-340-5p and its control, followed by OD+HG stimulation. Figure 4(a) demonstrated that miR-340-5p overexpression reduced ALP activity. Alizarin red staining result revealed that miR-340-5p elevation resulted in reduced calcium deposition (Figure 4(b)). Moreover, immunoblotting assay results showed that miR-340-5p down-regulated the expressions of OCN, collagen-I, and RUNX2 (Figure 4(c)). These findings further support the notion that miR-340-5p loss contributed to osteoblast differentiation under HG conditions.

### 3.5. RUNX2 was a target gene of miR-340-5p

**Figure 5.**
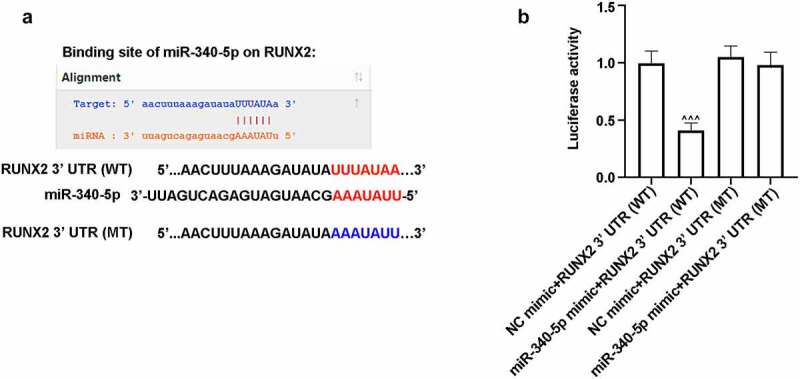
RUNX2 was a possible target gene of miR-340-5p. (a) The binding sequences of miR-340-5p and RUNX2 3ʹ UTR (WT or MT). (b) 293 T cells were co-transfected with miR-340-5p mimic or NC mimic and RUNX2 3ʹ UTR (WT) or RUNX2 3ʹ UTR (MT) for 48 h. After co-transfection, cells were lysed, and the luciferase activity was evaluated by the kit. Results were presented as means ± SD (N = 3 in each group). ^^^*P* < 0.001 versus miR-340-5p mimic+RUNX2 3ʹ UTR (MT) group. WT, wild type; MT, mutant type; UTR, untranslated region

TargetScanHuman website predicted the interaction of miR-340-5p and RUNX2, and Figure 5(a) presented the possible binding sequences of miR-340-5p with RUNX2 3ʹ UTR (WT or MT). To further verify the relationship between miR-340-5p and RUNX2, luciferae assay was carried out. As shown in Figure 5(b), the luciferase activity was obviously reduced in miR-340-5p mimic+RUNX2 3ʹ UTR (WT) group, as compared to miR-340-5p mimic+RUNX2 3ʹ UTR (MT) group, demonstrating that RUNX2 may be the target gene of miR-340-5p.

*3.6. MiR-340-5p loss facilitated osteogenic differentiation through regulation of RUNX2 in MC3T3-E1 cells*

**Figure 6.**
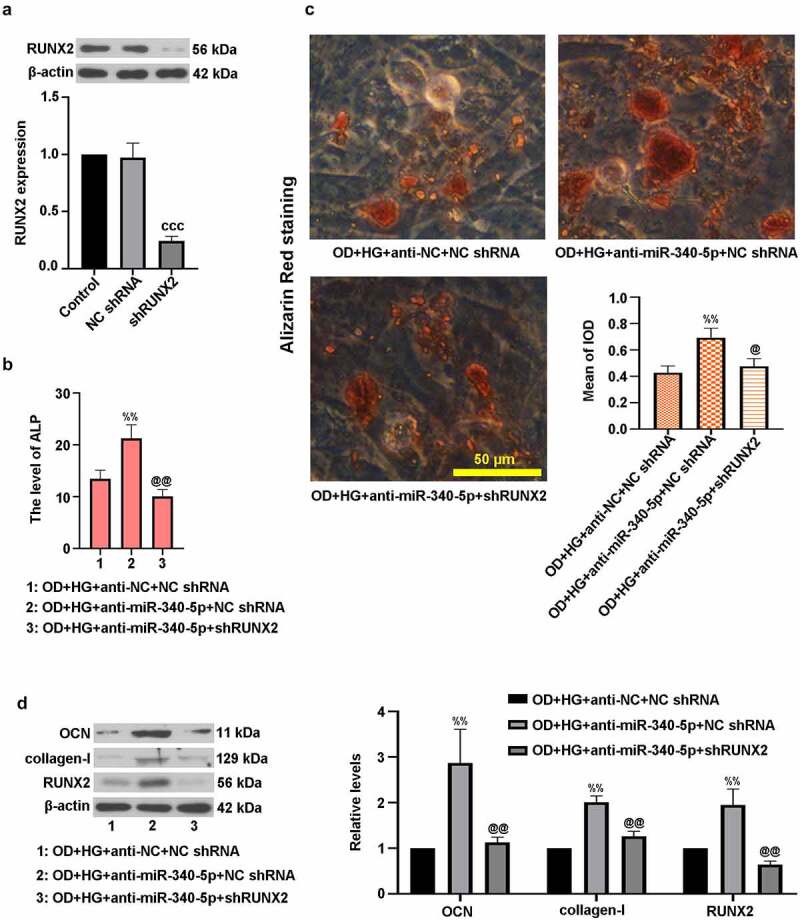
MiR-340-5p facilitated osteogenic differentiation through regulation of RUNX2 in MC3T3-E1 cells. (a) Cells were infected with lentivirus-based shRUNX2 or NC shRNA for 72 h. The protein expression of RUNX2 was detected by immunoblotting. (b) Cells were co-infected with anti-miR-340-5p or anti-NC and shRUNX2 or NC shRNA for 72 h. Subsequently, osteoblast differentiation was induced, and cells were treated with HG for 14 days. ALP activity was measured with the kit. (c) Treated cells were stained with Alizarin Red and quantified accordingly. Scale bar = 50 μm. (d) OCN, collagen-I, and RUNX2 protein levels were evaluated with immunoblotting. Data were represented as means ± SD (N = 3 per group). ^ccc^*P* < 0.001 versus NC shRNA; ^%%^*P* < 0.01 versus OD+HG+anti-NC+NC shRNA group; ^@^*P* < 0.05 and ^@@^*P* < 0.01 versus OD+HG+anti-miR-340-5p+NC shRNA group

To figure out the impact of RUNX2 on miR-340-5p-mediated osteoblast differentiation, we silenced RUNX2 in MC3T3-E1 cells, which was confirmed using immunoblotting (Figure 6(a)). Besides, cells were co-infected with anti-miR-340-5p and shRUNX2, and then treated with OD and HG. Figure 6(b) exhibited a decrease of ALP level in OD+HG+anti-miR-340-5p+shRUNX2 group. Inhibition of RUNX2 lowered calcium deposition in MC3T3-E1 cells (Figure 6(c)). Importantly, the decrease of OCN, collagen-I, and RUNX2 protein expressions was induced by RUNX2 down-regulation (Figure 6(d)). These findings showed that inhibition of miR-340-5p expression promoted osteoblast differentiation via increasing RUNX2 level in MC3T3-E1 cells.

## 4. Discussion

DOP is a kind of secondary osteoporosis [20], and its molecular mechanism remains unclear. In the present study, we established the rat model of DOP. As expected, the body weight of rats was lower accompanied by the increase of fasting blood glucose level and the decrease of insulin content as well as BMD in STZ+OVX (DOP) group, which was similar to a previous report [6]. There was a significant reduction of ALP level in the DOP rat model. It was also shown that the level of miR-340-5p was up-regulated while the protein expression of RUNX2 was down-regulated in femur tissues of DOP rats. These findings confirmed that DOP rat model was successfully developed in this study, and indicated the potential role of miR-340-5p in DOP.

Osteoblast dysfunction, one of the crucial mechanisms, results in osteoporosis [9]. Osteoblasts are closely correlated with bone formation and bone remodeling [21]. It has been reported that osteoblast differentiation depends upon the expression of RUNX2, an important regulator in bone formation [22]. In this study, MC3T3-E1 cells after OD and HG treatment was employed as the cell model of DOP. Our results indicated that HG elevated miR-340-5p level but lowered RUNX2 expression in OD-administrated cells, which was consistent with that of the *in vivo* studies. In addition, luciferase assay demonstrated that RUNX2 was a possible target gene of miR-340-5p. Data analysis showed that co-infection with anti-miR-340-5p and shRUNX2 reduced the level of RUNX2, contrary to that down-regulation of miR-340-5p enhanced RUNX2 expression. This implies that RUNX2 may play a regulatory role in osteoblast differentiation initiated by miR-340-5p. To the best of our knowledge, the finding that miR-340-5p reduction facilitated osteoblast differentiation in MC3T3-E1 cells via targeting RUNX2 has not been reported so far.

OCN and collagen-I are hallmarks of osteoblast differentiation, which can directly reflect the process of differentiation [15]. Here, the protein levels of OCN and collagen-I were evaluated by immunoblotting. The *in vivo* studies revealed lowered levels of these osteoblast differentiation markers in model group. What is more, the *in vitro* investigations showed that miR-340-5p inhibition elevated HG-induced decreased OCN, collagen-I and RUNX2 expressions, which was for the first time discovered. It is well-known that ALP activity is a critical marker for differentiation, especially in osteoblast differentiation, concomitant with the occurrence of extracellular matrix (ECM) mineralization [15]. In the current study, the increase of ALP activity and calcium deposition was observed in the process of osteoblast differentiation, whereas HG reversed these changes, consistent with earlier researches [2,23]. Subsequently, osteoblast differentiation was enhanced by down-regulation of miR-340-5p in HG-administrated cells via increasing ALP level and mineralization. It was also proven that RUNX2 possibly participated in regulation of miR-340-5p inhibition-triggered osteoblast differentiation in MC3T3-E1 cells. Similar results were discovered in the previous work [16]. These findings suggested that miR-340-5p deletion contributed to the recovery of osteoblast differentiation through up-regulating RUNX2.

RUNX2 has been regarded as a vital mediator of multiple signaling pathways associated with controlling osteoblast differentiation such as transforming growth factor-β (TGF-β)/bone morphogenetic proteins (BMP), MAPK, and Wnt/β-catenin pathways [5,13,16]. As reported by Wang et al., lncRNA KCNQ1OT1 modulated osteoblast differentiation of BMSCs through sponging miR-214 by the activation of BMP2/Smad signaling pathway [24]. Furthermore, β-catenin is considered a pivotal molecule intimately related to osteogenesis [4], and it goes into the nucleus to induce the activation of downstream factors, which has a key role in cell proliferation and differentiation [25]. Wnt and Akt signaling pathways are the primary pathways that have an influence on bone metabolism and calcium homeostasis [9]. As such, the effects of miR-340-5p on these signaling pathways are worthy of further research. Though in this study we initially found a new molecular mechanism of miR-340-5p/RUNX2 axis in modulating osteoblast differentiation in MC3T3-E1 cells, further studying the pathogenesis of DOP and its underlying mechanisms is very necessary. The present study mainly focused on *in vitro* studies to explore the role of miR-340-5p in osteoblast differentiation and our results showed that down-regulation of miR-340-5p promoted osteoblast differentiation of MC3T3-E1 cells. Since the animal model is more reference significance than the cell model, the *in vivo* investigations about the effect of miR-340-5p on the development of DOP should be conducted in the future.

## Conclusions

Collectively, our study revealed that inhibition of miR-340-5p promoted osteoblast differentiation by targeting RUNX2 through the increase of ALP activity, calcium deposition, and pro-osteogenic differentiation factors OCN and collagen-I in MC3T3-E1 cells. This suggested that miR-340-5p/RUNX2 axis may be a novel therapeutic target for the treatment of DOP.

## Supplementary Material

Supplemental Material

## Data Availability

The data used to support the findings of this study are included within the article.

## Highlights

- Inhibition of miR-340-5p enhanced alkaline phosphatase (ALP) level, calcium deposition, and osteocalcin (OCN), collagen-I and RUNX2 expressions.
- RUNX2 may be a target gene of miR-340-5p.
- RUNX2 down-regulation abolished the effects of miR-340-5p on osteogenic differentiation.
