# Supplementary figures and images for: Down-regulation of miR-340-5p promoted osteogenic differentiation through regulation of runt-related transcription factor-2 (RUNX2) in MC3T3-E1 cells
Source: Bioengineered. 2021 Apr 5;12(1):1126–37. doi: 10.1080/21655979.2021.1905259 (PMC8291863; doi:10.1080/21655979.2021.1905259)

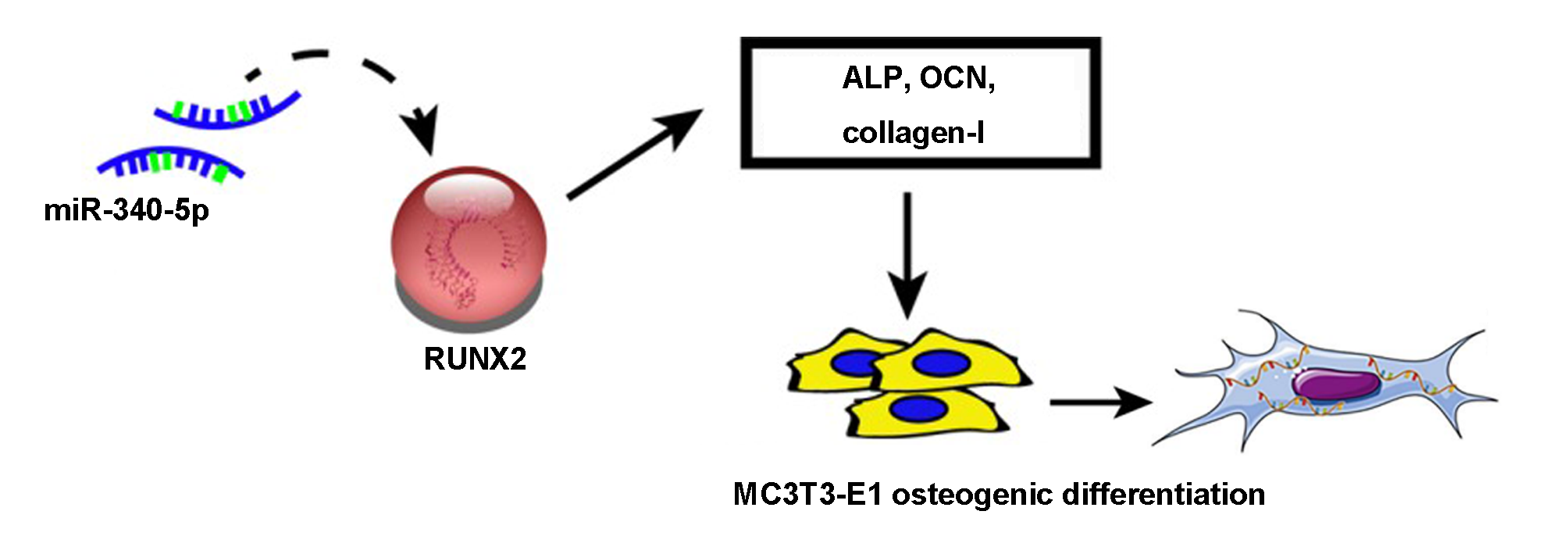

Supplement: Supplemental Material [file KBIE_A_1905259_SM1144.tif]
